# Highly Soluble Mussel Foot Protein Enhances Antioxidant Defense and Cytoprotection via PI3K/Akt and Nrf2/HO-1 Pathways

**DOI:** 10.3390/antiox14060644

**Published:** 2025-05-27

**Authors:** Na Li, Jiren Xu, Boheng Liu, Jeevithan Elango, Wenhui Wu

**Affiliations:** 1 Department of Marine Pharmacology, College of Food Science and Technology, Shanghai Ocean University, Shanghai 201306, China; d220300085@st.shou.edu.cn (N.L.); m240451270@st.shou.edu.cn (J.X.); 2334227@st.shou.edu.cn (B.L.); 2 Department of Biomaterials Engineering, Faculty of Health Sciences, UCAM-Universidad Católica San Antonio de Murcia, Guadalupe, 30107 Murcia, Spain; 3 Marine Biomedical Science and Technology Innovation Platform of Lin-gang Special Area, Shanghai 201306, China; 4 Putuo Branch of International Combined Research Center for Marine Biological Sciences, Zhoushan 316104, China

**Keywords:** mussel foot protein, antioxidant activity, cell protective effect, signaling pathways

## Abstract

Mussel foot protein is a bioadhesive protein with potential biomedical applications, but its limited solubility and poor biological stability hinder its widespread use. In this study, highly soluble mussel foot protein (HMFP) was successfully extracted using a stepwise selective enzymatic digestion method, with a molecular weight in the range of 11–17 kDa. Furthermore, a dual-functional polyethylene glycol (PEG) derivative of HMFP, designated HMFP-PEG, was synthesized. FTIR analysis confirmed the successful modification of HMFP with PEG, while TGA analysis and SEM observations demonstrated that PEG modification significantly enhanced the stability of the protein. Both HMFP and HMFP-PEG effectively scavenged free radicals, enhanced antioxidant enzyme activity, and reduced MDA levels. Additionally, they activated the PI3K/Akt and Nrf2/HO-1 signaling pathways, inhibiting H_2_O_2_-induced cell apoptosis. Notably, HMFP-PEG exhibited superior antioxidant and cell-protective effects compared to HMFP, suggesting that PEG modification improves the functional stability of the protein. This study provides theoretical support for the potential application of HMFP in the biomedical and tissue engineering fields.

## 1. Introduction

Oxidative stress, induced by reactive oxygen species (ROS) such as hydrogen peroxide (H_2_O_2_), is a significant factor contributing to cellular dysfunction, including inflammation [1], apoptosis [2], and tissue injury [3]. Specifically, oxidative stress plays a critical role in the pathogenesis of various diseases, such as cardiovascular diseases [4], neurodegenerative diseases [5], and cancer [6,7]. Fibroblasts, the primary cells in connective tissue, are particularly sensitive to oxidative injury, which may impair their ability to maintain tissue integrity and promote the onset of chronic inflammation [8,9,10]. Therefore, exploring potential therapeutic agents that can protect fibroblasts from oxidative stress-induced injury is of substantial clinical importance.

Mussels, widely distributed in marine environments, possess unique biological functions [11,12,13]. Mussel foot proteins (MFPs) are natural high-molecular-weight proteins, primarily secreted by mussels as their main adhesive proteins, and have shown significant biomedical potential [14,15,16]. The foot proteins of *Mytilus galloprovincialis* are primarily composed of specialized proteins that exhibit excellent adhesive properties, biodegradability, and biocompatibility, making them useful in wound healing and drug delivery applications [17,18,19]. MFPs are particularly rich in DOPA (3,4-dihydroxyphenylalanine) residues, which exhibit strong antioxidant properties [20,21,22]. DOPA has a powerful free radical scavenging ability, effectively mitigating oxidative stress-induced cellular injury [23,24]. As a marine natural product, mussel proteins have found application in bioadhesion and are increasingly attracting biomedical researchers due to their antioxidant and cell-protective characteristics.

Despite the significant antioxidant potential of mussel foot proteins, their poor solubility and low biological stability limit their widespread use in vivo [25]. Therefore, improving the solubility of these proteins to enhance their bioactivity is key to solving this issue. Current methods to improve protein solubility involve chemical modifications or the addition of excipients [25,26,27]. For example, increasing the polarity of protein molecules or employing enzymatic or acidic hydrolysis can improve the solubility to some extent [28,29]. However, existing technologies have not effectively addressed enhancements in protein solubility in water and improvements in its biological stability. Furthermore, most current approaches focus on the moisturizing properties of the proteins and their interactions with the skin [19,30], with less emphasis on their antioxidant effects.

In this study, we optimized the preparation process of *Mytilus galloprovincialis* mussel foot protein to obtain highly soluble mussel foot protein (HMFP) and further modified it with a dual-functional polyethylene glycol (PEG) derivative (HMFP-PEG). We evaluated its protective effects against H_2_O_2_-induced oxidative injury in fibroblasts, analyzing its impact on cell viability, apoptosis, antioxidant enzyme activity, and key signaling pathways. This research reveals the potential therapeutic value of HMFP and its derivatives in oxidative stress-related diseases and explores the application prospects of marine natural products, particularly mussel proteins, in cell protection and antioxidant therapy.

## 2. Materials and Methods

### 2.1. Materials and Reagents

The *Mytilus galloprovincialis* mussel foot byssus gland was provided by Rizhao Hengtai Agricultural Technology Co., Ltd. and Ruimei Aquaculture Co., Ltd (Rizhao, China). Neutral protease, pepsin, acetic acid, urea, and other reagents were purchased from the Sinopharm Group Chemical Reagent Co., Ltd. (Shanghai, China). PEG5000, NHS, maleimide, DTT, and Coomassie Brilliant Blue R-250 were obtained from the Sigma-Aldrich Corporation (St. Louis, MO, USA). PAGE gel preparation kits were purchased from the Epizyme Biomedical Technology Co., Ltd. (Shanghai, China). Antibodies for Bax, Bcl-2, PI3K, p-PI3K, Akt, p-Akt, Nrf2, HO-1, and GAPDH were sourced from Huabio (Hangzhou, China). The BCA protein assay kit was supplied by Abcam, Ltd. (Cambridge, UK). Unless otherwise specified, all reagents used in this study were of analytical grade.

### 2.2. Extraction of Highly Soluble Mussel Foot Protein (HMFP) from Foot Gland of Mytilus galloprovincialis

**Figure 1:**
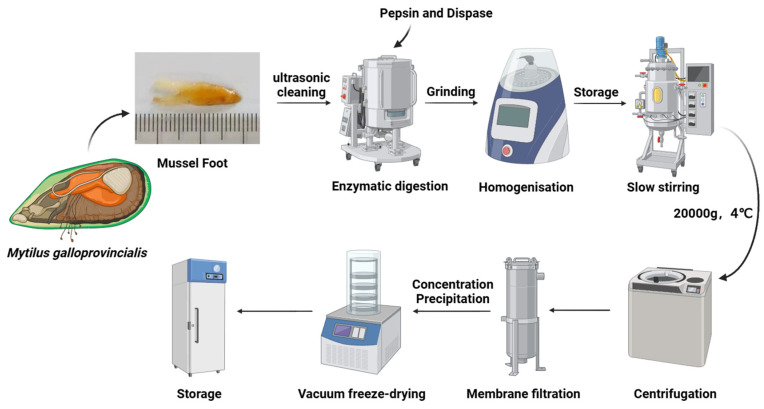
Detailed flowchart of the process of extracting highly soluble mussel foot protein (HMFP) from the foot gland of *Mytilus galloprovincialis*.

The mussel foot byssus protein was extracted from *Mytilus galloprovincialis* mussel foot byssus glands following a modified method based on the approach described by Lu et al. [31]. The frozen purple mussel foot byssus glands were thawed at −20 °C and placed in distilled water for ultrasonic cleaning (500 W, 45 min) to remove pigments and impurities. Subsequently, enzymatic hydrolysis was carried out using neutral protease, followed by pepsin under acidic conditions. The reaction was conducted in a thermostatic water bath with gentle stirring to ensure homogeneity. Upon completion of digestion, the hydrolysate was washed thoroughly with distilled water to remove residual enzymes and impurities. After enzymatic digestion, the foot byssus was minced using a meat grinder and further homogenized using a colloid mill. To disrupt protein–protein interactions, a solution of 8 M urea–5% acetic acid (*w*/*v* = 1:2) was added. The homogenate was then transferred to a high-level tank and gently stirred at 10–30 rpm for 30 min. The mixture was centrifuged at 20,000× *g* for 45 min at 4 °C, and the supernatant was collected as a crude protein extract. The crude protein extract was filtered through a 200 kDa ceramic membrane (TAMI Industries, Nyons, France) (3.0 bar feed pressure, 2.0 bar permeate pressure, 45 °C) to remove impurities. Urea was removed by chemical precipitation (ammonium sulfate) combined with centrifugation or by nanofiltration (10 kDa), concentrating the solution to 1/10th of the original volume. Finally, the protein extract was lyophilized to obtain the highly soluble mussel foot protein (HMFP). Figure 1 illustrates the detailed process of extracting the mussel foot byssus protein.

### 2.3. Gel Filtration Chromatography Using Sephadex G-50

Purification was carried out on an AKTA pure system (Inscinstech, Suzhou, China) using a Sephadex G−50 column (1.6 cm × 70 cm) (TianDiRenHe Biotechnology Co., Ltd., Beijing, China). The column was equilibrated and eluted with 50 mmol/L phosphate buffer (pH=7.0) at a flow rate of 0.5 mL/min. A 1 mL sample containing 10 mg of protein was loaded onto the column. Elution was monitored at 280 nm, and fractions corresponding to the main peak were collected for further analysis.

### 2.4. SDS-PAGE 

The HMFP sample was dissolved in distilled water and mixed with SDS−PAGE loading buffer in a 4:1 ratio. After thorough mixing, the sample was heated in a 95 °C water bath for 3–5 min to ensure complete protein denaturation. The processed sample was then allowed to cool to room temperature and briefly centrifuged at 1000× *g*. Protein samples and a protein molecular weight marker (with a molecular weight range from 10 kDa to 250 kDa) were loaded onto a 4.5% stacking gel and a 12.5% separating gel, with 10 μL of the sample loaded into each well. Electrophoresis was performed at a constant voltage of 180 V for 50 min to effectively separate the HMFP samples. After electrophoresis, the gel was stained with Coomassie Brilliant Blue for 30 min and then destained. Finally, the gel was imaged using a gel imaging system, and the results were recorded for the analysis of the protein molecular weight and band characteristics.

### 2.5. Determination of DOPA Content in HMFP

We referred to the method of J. H. Waite and colleagues, with slight modifications [32]. The procedure is as follows: dissolve 0.025 g of HMFP in 0.012 mol·L^−1^ hydrochloric acid, supplementing the volume to 50 mL to obtain a 0.5 mg·mL^−1^ protein solution. Transfer 1 mL each of the standard solutions and the protein solution into separate test tubes. Add 0.5 mL of 0.516 mol·L^−1^ hydrochloric acid to each tube, followed by 1.5 mL of diazotization reagent. After 5 min, add 2 mL of 1 mol·L^−1^ sodium hydroxide, mix thoroughly, and measure the absorbance at 500 nm.

### 2.6. PEGylation of Highly Soluble Mussel Foot Protein Using NHS-PEG-MAL

The PEGylation of HMFP was carried out using N−hydroxysuccinimide–maleimide–bifunctional polyethylene glycol (NHS−PEG−MAL), and, after reviewing the relevant literature, it was decided to select PEG5000 nin order to balance solubility and steric effects [33]. Initially, small-molecule impurities in HMFP were removed via dialysis (MWCO 5 kDa) to ensure protein purity. The purified HMFP was then dissolved in phosphate-buffered saline (PBS, pH = 7.4) at a final concentration of 3–5 mg/mL. Subsequently, NHS-PEG-MAL was added dropwise to the HMFP solution under continuous stirring at a 2:1 molar ratio (PEG–HMFP). The reaction proceeded at 4 °C for 48 h to allow for efficient conjugation, where the NHS group selectively formed amide bonds with lysine residues, and the maleimide group covalently reacted with cysteine side chains. Upon completion, unreacted PEG was removed by dialysis (MWCO 6 kDa) to ensure the purity of the PEGylated product. Finally, the successful PEGylation and the structural characteristics of HMFP-PEG were confirmed by Fourier transform infrared spectroscopy (FTIR) (Nicolet iS10, Thermo Fisher Scientific, Waltham, MA, USA).

### 2.7. FTIR Analysis of HMFP

The extracted HMFP and HMFP-PEG were dried and ground into a fine powder and then mixed with an appropriate amount of dried KBr powder to prepare a translucent or transparent sample pellet. Subsequently, FTIR spectroscopy was performed to analyze the sample. The scanning wavenumber range was set from 4000 to 400 cm^−1^, with a resolution of 4 cm^−1^. The absorption peaks in various wavenumber regions were analyzed to reveal the functional groups and structural characteristics of the protein.

### 2.8. Thermogravimetric Analysis of HMFP and HMFP-PEG

Precise amounts of dried HMFP and HMFP-PEG (approximately 5–10 mg) were placed in the sample pan of a thermogravimetric analyzer (TGA) (TGA Q500, TA Instruments, New Castle, DE, USA). The heating rate was set to 10 °C/min, and the test was conducted under a nitrogen atmosphere. The temperature range for the analysis was set from 50 °C to 500 °C. The thermogravimetric (TG) curve was obtained by monitoring the mass change of the sample as a function of the temperature. In addition, derivative thermogravimetry (DTG) analysis, which calculates the rate of mass loss as a function of the temperature, was used to more precisely identify the temperatures corresponding to thermal decomposition events. Based on the different stages of mass loss and their derivatives, the thermal stability, decomposition temperature, and other thermal behavior characteristics of the sample were analyzed.

### 2.9. Morphological Characterization of HMFP and HMFP-PEG via SEM

Suitable amounts of HMFP and HMFP-PEG were placed on the sample stage and subjected to freeze-drying to remove moisture while preserving their structural stability. The samples were then coated with a thin layer of metal to enhance the conductivity. Subsequently, SEM (JSM-IT500, JEOL Ltd., Tokyo, Japan) was performed with an accelerating voltage of 2 kV to examine the surface morphology. High-resolution images were obtained, and the microstructural characteristics of the samples were analyzed based on the acquired images.

### 2.10. Cell Activity Assay

The protective effects of HMFP and its derivative on H_2_O_2_-induced fibroblast injury were assessed using the CCK-8 assay. Fibroblasts were seeded in 96-well plates at a density of 5 × 10^4^ cells/mL and incubated for 24 h. Different concentrations of HMFP and its derivative were added to the cells, followed by 6 h incubation. Subsequently, 300 μM H_2_O_2_ was added, and the cells were cultured for an additional 12 h. Afterward, 10 μL of CCK-8 reagent was added to each well, and the cells were incubated for 1 h. The absorbance at 450 nm was measured to determine the OD, and the cell viability was calculated.

### 2.11. Evaluation of In Vitro Antioxidant Activity of HMFP and HMFP-PEG Using DPPH Radical Scavenging Assay

The in vitro antioxidant activity of HMFP and HMFP-PEG was evaluated using the DPPH radical scavenging assay. Solutions of HMFP and its derivatives at different concentrations were mixed with an equal volume of 0.2 mM DPPH ethanol solution and reacted in the dark for 30 min. The absorbance at 517 nm was measured, and the DPPH radical scavenging rate was calculated.

### 2.12. Assessment of Antioxidant Enzyme Activity

L-929 cells were seeded at a density of 1 × 10^6^ cells in 6-well plates and cultured until 80% confluence. The culture medium was then replaced with serum-free medium, and different concentrations of HMFP and HMFP-PEG were added to the cells for 24 h. Subsequently, 300 μM H_2_O_2_ was added to each group to induce oxidative injury, and the cells were incubated for an additional 4 h to establish the oxidative injury model. The control group was cultured with medium only, and the model group did not receive protein samples. After the treatment, cells were washed with PBS and collected. Following the instructions provided by the reagent kits (Beyotime, Shanghai, China), the activity of SOD, CAT, and GSH-Px and the content of MDA were measured.

### 2.13. Assessment of Cell Apoptosis

To evaluate cell apoptosis, L-929 cells were treated under different conditions, followed by digestion with trypsin and resuspension in PBS. According to the instructions provided by the reagent kit (Beyotime, Shanghai, China), 5 μL of Annexin V-FITC and 10 μL of PI staining solution were added, and the cells were incubated in the dark for 15 min. Subsequently, apoptosis was assessed using a BD FACS Celesta flow cytometer (BD Biosciences, New York, NY, USA), and the apoptosis rate was analyzed using the FlowJo software (version 10.8.1).

### 2.14. Western Blotting Analysis

After treatment, the total protein was extracted using RIPA lysis buffer, and the protein concentration was determined by the BCA method. Equal amounts of protein were subjected to SDS-PAGE electrophoresis and transferred to a PVDF membrane, which was then blocked with 5% non-fat milk for 1 h. The membrane was incubated overnight at 4 °C with the following primary antibodies: Bax, Bcl-2, PI3K, p-PI3K, Akt, p-Akt, Nrf2, and HO-1 (1:1000), with GAPDH as the internal control (1:5000). After washing, the HRP-conjugated secondary antibody (1:5000) was added and the sample incubated at room temperature for 1 h. Protein bands were visualized using an ECL chemiluminescent substrate, and images were captured with an Amersham Imager 600 system. The relative expression levels of the proteins were quantified by analyzing the grayscale values of the bands using the ImageJ software (version 1.54p).

### 2.15. Statistical Analysis

Data are presented as the mean ± standard deviation (SD) from at least three independent experiments. Duncan’s multiple range test and one-way ANOVA were used to evaluate the significance of differences between group means, with the statistical analysis performed using SPSS 17.0 (SPSS, Inc., Chicago, IL, USA). Differences were considered statistically significant when * *p* < 0.05 (** *p* < 0.01, *** *p* < 0.001, ^#^ *p* < 0.05, ^##^ *p* < 0.01, and ^###^ *p* < 0.001).

## 3. Results

### 3.1. SDS-PAGE Analysis of HMFP

**Figure 2:**
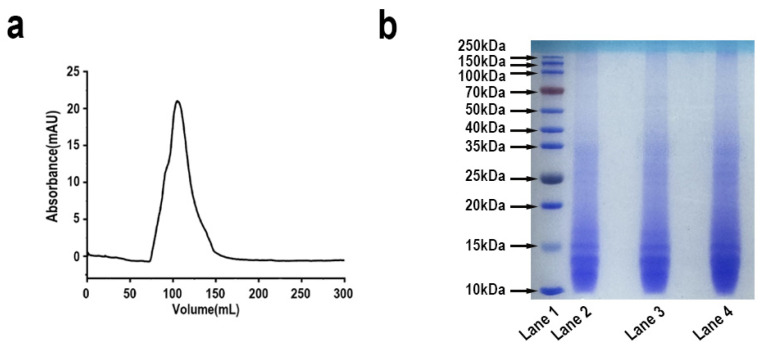
(**a**) Gel filtration chromatogram of HMFP on a Sephadex G-50 column. (**b**) SDS-PAGE separation (12.5%) of HMFP, stained with Coomassie Brilliant Blue R-250, followed by transfer to decolorization solution (ethanol–acetic acid: VH_2_O = 2:1:7). Lane 1: molecular weight markers (10−250 kDa); Lane 2: HMFP (0.5 mg/mL); Lane 3: HMFP (1 mg/mL); Lane 4: HMFP (1.5 mg/mL).

In this study, a stepwise selective enzymatic hydrolysis method was employed to effectively extract highly soluble mussel foot protein (HMFP) from mussel foot tissue. The process involved the gentle action of neutral protease and pepsin, which successfully removed high-molecular-weight proteins (Mfp-1, Mfp-2, and Mfp-4) from the byssal proteins, while retaining the structural integrity of the mussel foot gland. The Sephadex G-50 gel filtration chromatogram (Figure 2a) displayed a single sharp peak, indicating high molecular size homogeneity and relatively high purity. The resulting HMFP exhibited excellent solubility in distilled water, reaching up to 100 mg/mL, with DOPA content of approximately 11 mg/mL. The SDS-PAGE analysis (Figure 2b) showed major protein bands in the range of 11–17 kDa, corresponding to Mfp-3 and Mfp-5 isoforms. A faint band near ~35 kDa was also observed, which may represent dimeric or aggregated forms of HMFP. The isolated highly soluble fraction was thus designated as HMFP for subsequent characterization and application studies.

### 3.2. FTIR and Secondary Structure Analysis of PEG-Modified Mussel Foot Protein

**Figure 3:**
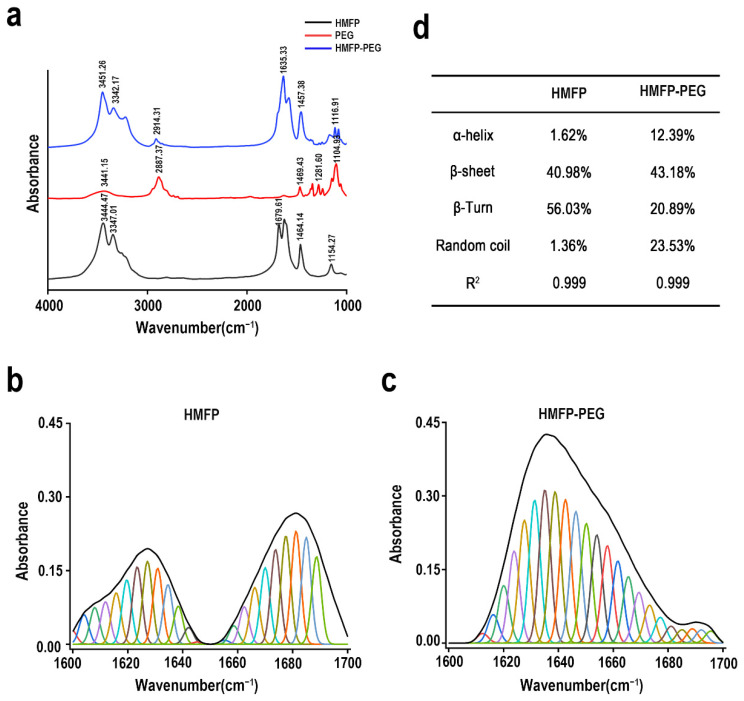
Secondary structure characterization of HMFP and HMFP-PEG. (**a**) FTIR spectra of HMFP and HMFP-PEG. (**b**,**c**) Deconvoluted amide I band (1600–1700 cm^−1^) indicating secondary structure components of HMFP and HMFP-PEG. (**d**) Quantitative analysis of α-helix, β-sheet, β-turn, and random coil content derived from spectra in (**b**,**c**).

The FTIR spectroscopy analysis confirmed that the bifunctional PEG was successfully conjugated to HMFP (Figure 3a). The characteristic absorption peaks of PEG, such as the C-O-C stretching vibration peak (around 1100 cm^−1^) and the absorption peak of the -OH group (3200–3500 cm^−1^), were observed in the modified infrared spectrum, indicating the successful attachment of PEG to the protein. Additionally, the absorption peaks associated with the protein itself (such as 1640 cm^−1^ and 1540 cm^−1^) underwent noticeable shifts, suggesting that the PEG modification impacted the structural conformation of HMFP. Secondary structure analysis (Figure 3b–d) revealed that, following PEG modification, the α-helix content of HMFP significantly increased from 1.62% to 12.39%. In contrast, the β-sheet content decreased from 40.98% to 43.18%, the β-turn content dropped from 56.03% to 20.89%, and the random coil content increased from 1.36% to 23.53%. These changes suggest that PEG modification altered the conformation of HMFP, potentially promoting a more relaxed and flexible structure in the dry state, although with a slight reduction in thermal stability and compactness.

### 3.3. Thermal Stability Analysis of HMFP and HMFP-PEG by TGA and DTG

**Figure 4:**
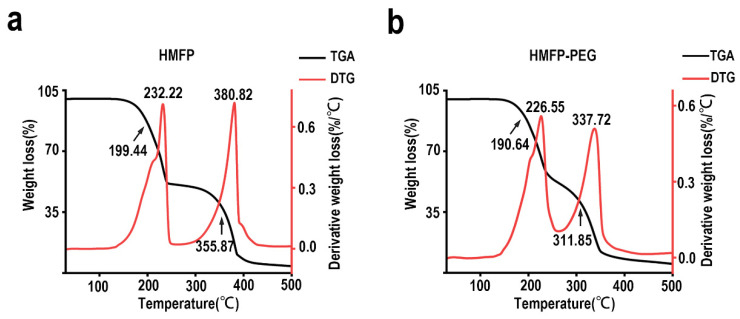
Thermal stability of HMFP and HMFP-PEG. (**a**) Thermogravimetric analysis (TGA) curves and (**b**) derivative thermogravimetric (DTG) curves showing thermal degradation profiles of HMFP and HMFP-PEG under nitrogen atmosphere.

The thermogravimetric analysis (TGA) and derivative thermogravimetry (DTG) analysis results revealed significant changes in the thermal stability of HMFP and its PEG-modified derivative (Figure 4). The TGA curve of HMFP showed two distinct mass loss points at 199.44 °C and 355.87 °C, with DTG peaks at 232.22 °C and 380.82 °C, respectively. These data suggest that the protein underwent different thermal degradation processes within these two temperature ranges. After PEG modification, the TGA characteristic points shifted to 190.64 °C and 311.85 °C, and the DTG peaks were observed at 226.55 °C and 337.72 °C, respectively. Compared to HMFP, the thermal degradation characteristics of HMFP-PEG in the low-temperature region (around 190 °C) and high-temperature region (around 311 °C) exhibited notable changes, indicating that PEG modification altered the thermal stability of the protein.

### 3.4. SEM Analysis of Dried-State Surface Morphology of HMFP and HMFP-PEG

**Figure 5:**
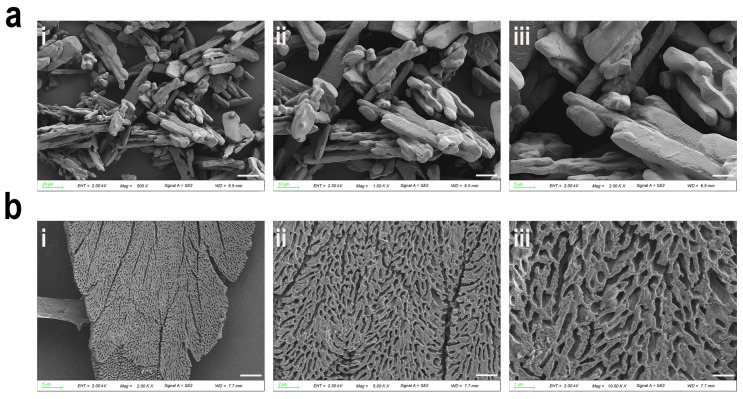
Surface morphology of freeze-dried HMFP and HMFP-PEG. SEM images of dried HMFP (**a**) ((**i**): 20 µm; (**ii**): 10 µm; (**iii**): 5 µm) and HMFP-PEG (**b**) ((**i**): 5 µm; (**ii**): 2 µm; (**iii**): 1 µm), illustrating the microstructural differences and surface textures after freeze-drying.

SEM observations revealed that HMFP exhibited a rod-like structure in its dry state, indicating strong protein–protein interactions (Figure 5). Upon PEGylation, the surface morphology underwent noticeable changes, displaying a more regular, flat, and strip-like structure with partial porosity. This morphological change may be attributed to alterations in the self-assembly process induced by PEG, particularly PEG’s enhancement of the protein’s hydrophilicity and disruption of hydrophobic interactions. Furthermore, the steric hindrance provided by PEG crosslinking may have impeded the close packing of the protein. NHS-PEG-MAL formed a crosslinked network through covalent modification, thereby altering the protein aggregation pattern. Although these structural features were observed under dried conditions, they suggest that PEG modification impacts the aggregation pattern and potentially enhances the solubility and colloidal stability of HMFP in solution.

### 3.5. Protective Effects of HMFP and HMFP-PEG Against H_2_O_2_-Induced Oxidative Stress in Fibroblasts

The extracted Mfp-3 and Mfp-5 proteins are closely related to antioxidant activity, primarily due to the high content of DOPA residues [34,35,36]. DOPA has strong antioxidant properties, capable of scavenging free radicals in the body and protecting cells from oxidative stress-induced injury [22,37]. Therefore, we further investigated the protective effects of HMFP and HMFP-PEG against hydrogen peroxide (H_2_O_2_)-induced fibroblast cell injury.

**Figure 6:**
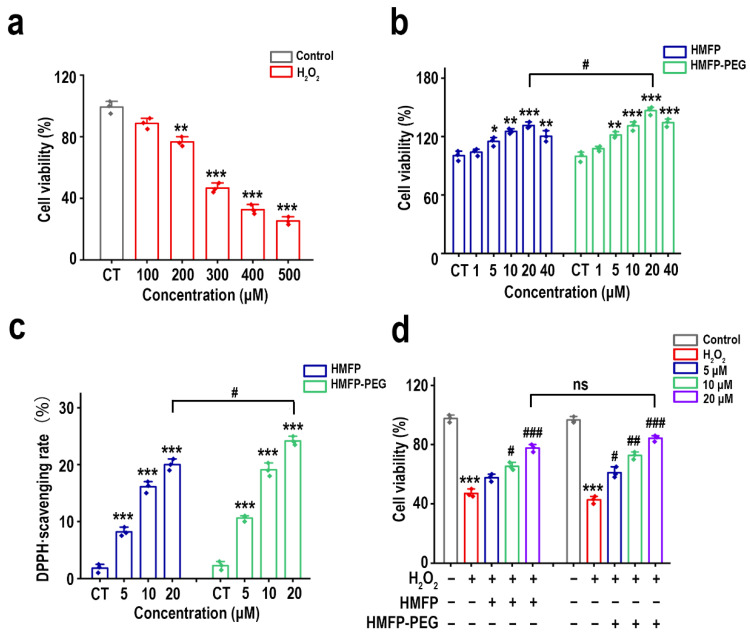
Evaluation of cytocompatibility and antioxidant protective effects of HMFP and HMFP-PEG. (**a**) Fibroblast viability after exposure to H_2_O_2_, establishing an oxidative damage model. (**b**) Effects of HMFP and HMFP-PEG on fibroblasts’ viability. (**c**) DPPH radical scavenging activity of HMFP and HMFP-PEG. (**d**) Cell viability under oxidative stress preconditioned with HMFP and HMFP-PEG. *, **, and *** represent *p* < 0.05, *p* < 0.01, and *p* < 0.001 compared to the control group, respectively; ^#^, ^##^, and ^###^ represent *p* < 0.05, *p* < 0.01, and *p* < 0.001 compared to the model group, respectively. ns indicates no significant difference.

In the H_2_O_2_-induced oxidative stress model, the cell viability significantly decreased with increasing H_2_O_2_ concentrations (Figure 6a). At a H_2_O_2_ concentration of 100 μM, the cell viability remained above 90%, showing no significant difference compared to the control group. At a concentration of 300 μM, the cell viability dropped to 55.68 ± 4.43%, approaching 50%, which was considered the optimal concentration for the oxidative stress model. Subsequently, different concentrations of HMFP and HMFP-PEG were applied to treat fibroblasts. The results showed that, within the concentration range of 1–20 μM, cell proliferation increased with increasing concentrations of HMFP and HMFP-PEG in a concentration-dependent manner (Figure 6b). However, when the concentration of HMFP and HMFP-PEG was 40 μM, although a significant difference was observed compared to the blank group, the cell viability decreased compared to 20 μM, showing a non-concentration-dependent pattern. Therefore, 5 μM, 10 μM, and 20 μM concentrations of HMFP and HMFP-PEG were selected for subsequent antioxidant activity studies. The DPPH assay results (Figure 6c) indicated that both HMFP and HMFP-PEG exhibited good antioxidant activity, with HMFP-PEG showing significantly enhanced antioxidant activity at 20 μM. Furthermore, when oxidative stress was preconditioned with HMFP and HMFP-PEG, the cell survival rate was positively correlated with their concentrations (Figure 6d).

### 3.6. The Effects of HMFP and HMFP-PEG on Antioxidant Enzymes and MDA in Oxidative Stress Fibroblasts

**Figure 7:**
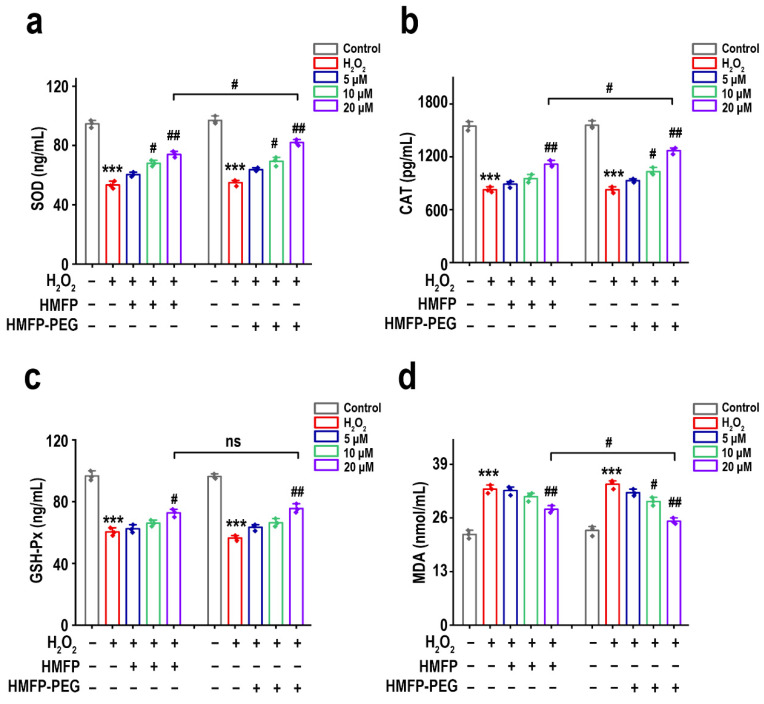
Effects of HMFP and HMFP-PEG on intracellular antioxidant enzyme activity. Levels of oxidative stress biomarkers in L-929 fibroblasts: (**a**) SOD activity, (**b**) CAT activity, (**c**) GSH-Px activity, and (**d**) MDA content after pre-treatment with HMFP and HMFP-PEG. *** represent *p* < 0.001 compared to the control group; ^#^, and ^##^ represent *p* < 0.05, and *p* < 0.01 compared to the model group, respectively. ns indicates no significant difference.

Cell injury induced by oxidative stress is often closely associated with changes in the activity of key antioxidant enzymes, including SOD [38], CAT [39], and GSH-Px [40,41], as well as the oxidation injury marker MDA [42,43]. The results (Figure 7) showed that, compared to the blank group, H_2_O_2_ induction significantly decreased the activity of SOD, CAT, and GSH-Px in fibroblasts, while the MDA level significantly increased, indicating oxidative injury to the cells. After pre-treatment with HMFP and HMFP-PEG, the activity of SOD, CAT, and GSH-Px significantly increased compared to the model group, in a concentration-dependent manner, with the most significant differences observed at the higher concentration (20 μM). Meanwhile, the MDA levels significantly decreased, further confirming the antioxidant effects of HMFP and HMFP-PEG. Notably, the HMFP-PEG group showed superior enhancements in SOD and CAT activity compared to the HMFP group, highlighting the unique advantage of PEG modification in enhancing the antioxidant capacity.

### 3.7. HMFP and HMFP-PEG Reduce Hydrogen Peroxide-Induced Apoptosis of Fibroblasts

**Figure 8:**
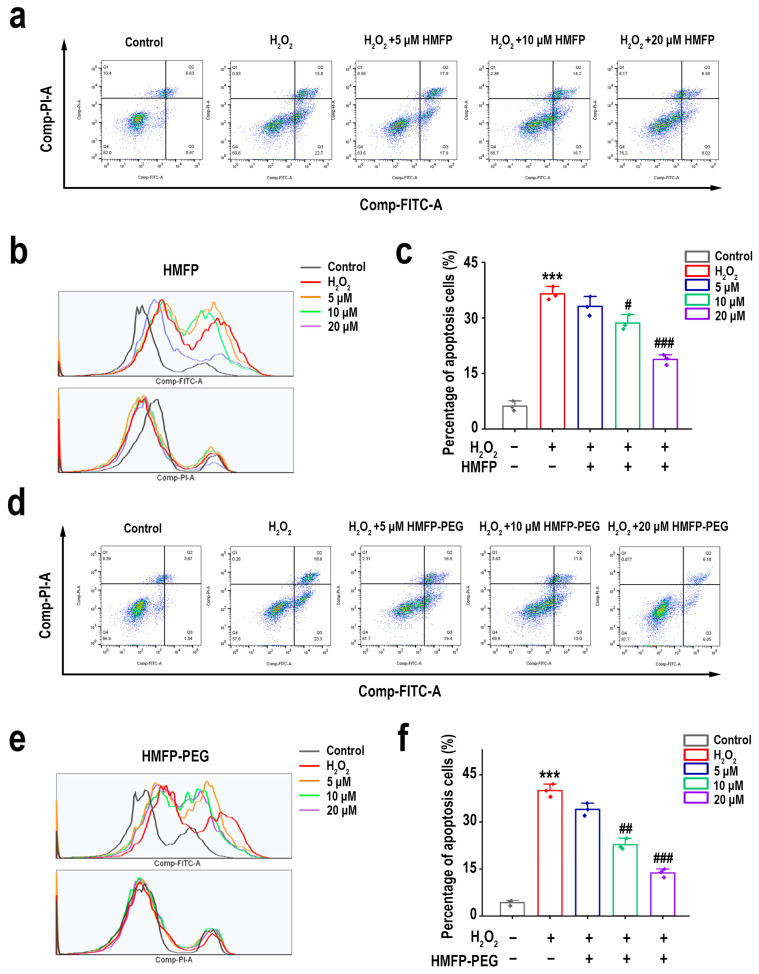
Apoptosis analysis by flow cytometry after oxidative stress and treatment. Flow-cytometric plots showing apoptotic cell populations in fibroblasts treated with HMFP (**a**–**c**) and HMFP-PEG (**d**–**f**), with or without oxidative stress. *** represent *p* < 0.001 compared to the control group; ^#^, ^##^, and ^###^ represent *p* < 0.05, *p* < 0.01, and *p* < 0.001 compared to the model group, respectively.

To further confirm the protective effects of HMFP and HMFP-PEG against H_2_O_2_-induced oxidative injury in fibroblasts, we assessed cell apoptosis using flow cytometry (Figure 8). As expected, compared to the blank group, H_2_O_2_ induction significantly increased the apoptosis rate, indicating that oxidative stress can induce fibroblast apoptosis. After pre-treatment with HMFP and HMFP-PEG, the total apoptosis rate significantly decreased in a concentration-dependent manner, suggesting that both treatments effectively inhibited H_2_O_2_-induced cell apoptosis, further confirming their protective effects against cell injury. Notably, at the medium concentration, HMFP-PEG (18.8%) significantly reduced the proportion of apoptotic cells compared to HMFP (22.36%), highlighting the superior protective effect of HMFP-PEG in reducing cell apoptosis.

### 3.8. Regulation of Bcl-2/Bax Ratio by HMFP and HMFP-PEG in H_2_O_2_-Induced Fibroblast Apoptosis

**Figure 9:**
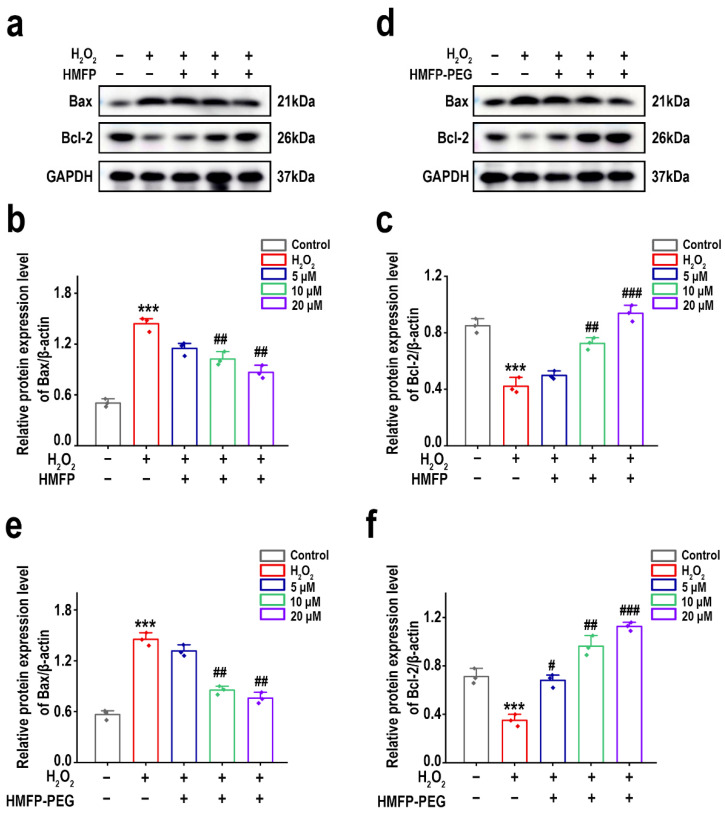
Western blot analysis of Bax and Bcl-2 expression in fibroblasts after pre-treatment with HMFP (**a**–**c**) and HMFP-PEG (**d**–**f**). All target protein levels were normalized to GAPDH and quantified using ImageJ. *** represent *p* < 0.001 compared to the control group; ^#^, ^##^, and ^###^ represent *p* < 0.05, *p* < 0.01, and *p* < 0.001 compared to the model group, respectively.

The Western blot (WB) results indicated significant changes in the expression levels of Bax and Bcl-2 across different treatment groups. As shown in Figure 9, compared to the blank group, H_2_O_2_ induction resulted in a marked increase in the expression of pro-apoptotic protein Bax and a significant decrease in the expression of anti-apoptotic protein Bcl-2. After pre-treatment with HMFP and HMFP-PEG, the expression of Bax significantly decreased, while the expression of Bcl-2 markedly increased, exhibiting a concentration-dependent effect. Notably, the HMFP-PEG group showed superior regulation of the Bcl-2/Bax ratio compared to the HMFP group, which may be related to its ability to improve the intracellular redox balance and enhance antioxidant enzyme activity.

### 3.9. HMFP and HMFP-PEG Activate the PI3K/Akt Signaling Pathway to Enhance Cell Proliferation

**Figure 10:**
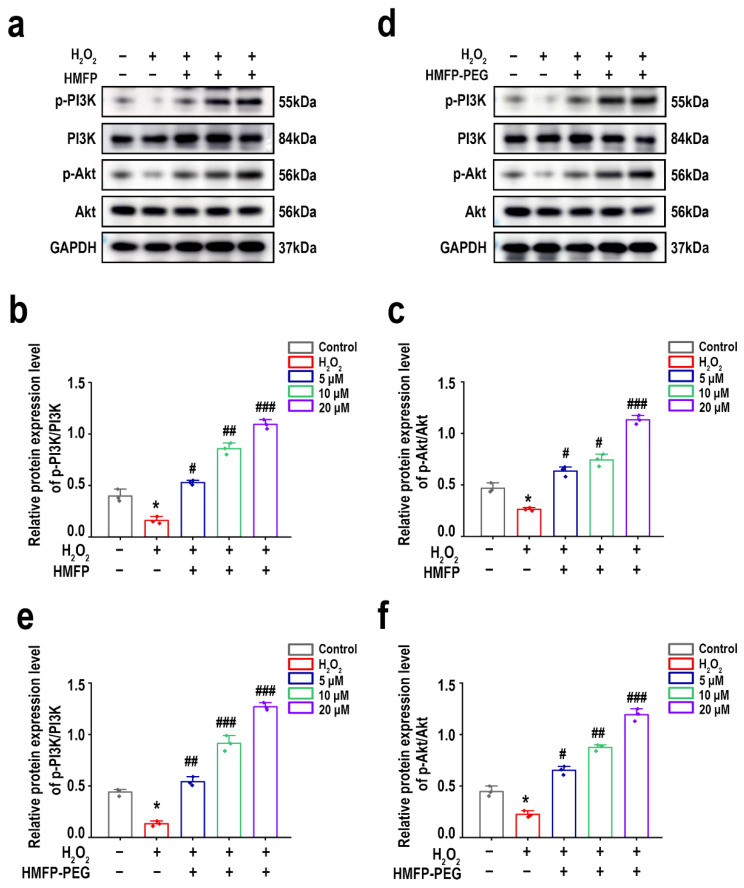
Western blot analysis of PI3K-Akt signaling pathway-related proteins in fibroblasts after pre-treatment with HMFP (**a**–**c**) and HMFP-PEG (**d**–**f**). All target protein levels were normalized to GAPDH and quantified using ImageJ. * represent *p* < 0.05 compared to the control group; ^#^, ^##^, and ^###^ represent *p* < 0.05, *p* < 0.01, and *p* < 0.001 compared to the model group, respectively.

The PI3K/Akt signaling pathway plays a crucial role in regulating cellular antioxidant defense mechanisms, thereby reducing oxidative stress injury and enhancing cell survival [44,45,46]. The Western blot (WB) results demonstrated significant differences in the expression levels of p-PI3K and p-Akt across the different treatment groups. As shown in Figure 10, compared to the blank group, H_2_O_2_ induction led to a substantial reduction in the expression of p-PI3K and p-Akt. After pre-treatment with HMFP and HMFP-PEG, the expression levels of p-PI3K and p-Akt significantly increased, showing a concentration-dependent effect, with a more pronounced enhancement observed in the HMFP-PEG group. Therefore, HMFP and HMFP-PEG may improve H_2_O_2_-induced oxidative stress injury by activating the PI3K/Akt signaling pathway, thereby enhancing the cellular antioxidant capacity.

### 3.10. Activation of Nrf2/HO-1 Signaling Pathway by HMFP and HMFP-PEG in Enhancing Antioxidant Defenses

**Figure 11:**
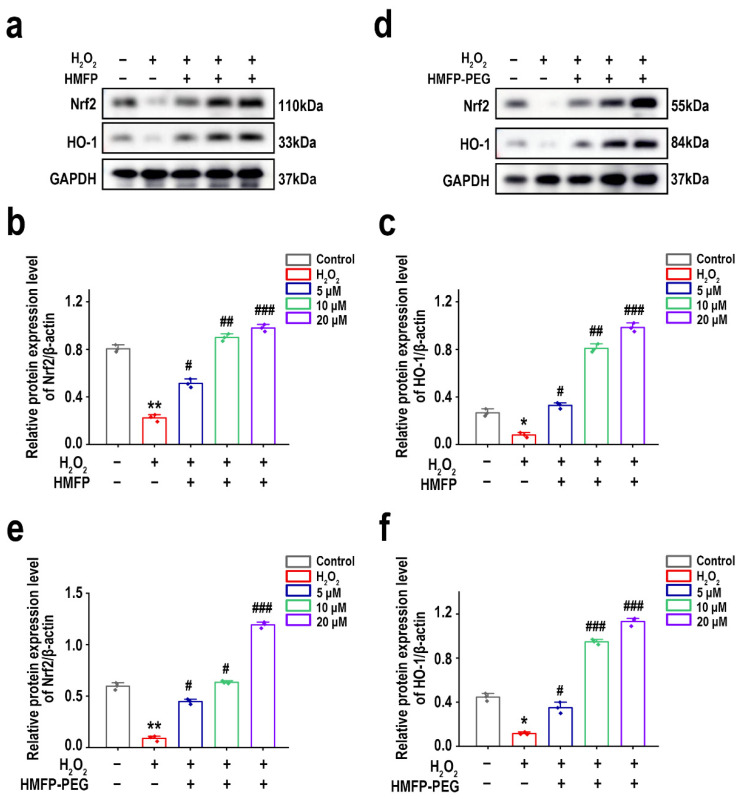
Western blot analysis of Nrf2 and HO-1 expression in fibroblasts after pre-treatment with HMFP (**a**–**c**) and HMFP-PEG (**d**–**f**). All target protein levels were normalized to GAPDH and quantified using ImageJ. *, and ** represent *p* < 0.05, and *p* < 0.01 compared to the control group, respectively; ^#^, ^##^, and ^###^ represent *p* < 0.05, *p* < 0.01, and *p* < 0.001 compared to the model group, respectively.

The Nrf2/HO-1 signaling pathway plays a crucial role in antioxidant stress responses [47,48,49]. The Western blot results (Figure 11) showed that, after pre-treatment with HMFP and HMFP-PEG, the expression levels of Nrf2 and HO-1 in fibroblasts were significantly increased, indicating that HMFP and HMFP-PEG may enhance the cells’ antioxidant capacity by activating the Nrf2/HO-1 signaling pathway. The HMFP-PEG group exhibited higher expression levels of Nrf2 and HO-1 compared to the HMFP group, suggesting that PEG modification may enhance its antioxidant effects.

## 4. Discussion

MFPs are natural polymers rich in DOPA residues, known for their excellent adhesive properties, biodegradability, and antioxidant characteristics [50,51,52]. These proteins have broad application potential in biomedical fields, such as wound healing and drug delivery [18,53,54]. Notably, mussel foot adhesive proteins are diverse, mainly including six components: MFP-1 to MFP-6 [55,56]. Among them, Mfp-3 and Mfp-5 have the highest DOPA content, reaching 25–30%, making them the most valuable components of mussel foot proteins [34,57,58]. In this study, we successfully prepared highly soluble mussel foot adhesive protein (HMFP) using a stepwise selective enzymatic hydrolysis method. This approach allows mussel proteins to dissolve in distilled water (Figure 1), effectively solving the solubility issue of mussel adhesive proteins. The Sephadex G-50 gel filtration chromatogram displayed a single sharp peak. Furthermore, our optimized extraction method removes the outer and middle layers of Mfp-1, Mfp-2, and Mfp-4, retaining the Mfp-3 and Mfp-5 components, which have high DOPA content and molecular weights in the range of 11–17 kDa (Figure 2).

MFPs are rich in DOPA, and their excellent adhesion and antioxidant properties primarily stem from the DOPA functional group [59,60]. However, under oxidative conditions, DOPA is prone to oxidation into quinones, which reduces its activity [61]. PEG modification can effectively inhibit DOPA auto-oxidation, improving its stability; reducing oxidative polymerization and preventing DOPA inactivation; and decreasing the impacts of external oxidants through the solvent shielding effect [62]. Among the modifications, bifunctional PEG (NHS-PEG-MAL) can covalently bond with HMFP through its two different reactive groups at both ends. Compared to monofunctional PEG modification, NHS-PEG-MAL has the advantage of forming a more effective PEG protective layer, which can significantly reduce oxidative injury, stabilize DOPA activity, enhance solubility, and prevent protein aggregation. This modification, therefore, increases the potential of HMFP in oxidative stress protection and enhances its biomedical value.

The Fourier transform infrared spectroscopy (FTIR) analysis confirmed the successful modification of HMFP by NHS-PEG-MAL (Figure 3). The modified spectra clearly showed PEG’s characteristic absorption peaks, such as the C-O-C stretching vibration peak around 1100 cm^−1^ and the -OH group absorption peak in the 3200–3500 cm^−1^ range, indicating the successful incorporation of PEG into the protein. Additionally, the absorption peaks of the protein itself (such as 1640 cm^−1^ and 1540 cm^−1^) underwent shifts, suggesting that the PEG modification had an impact on the structure of HMFP. Secondary structure analysis further revealed that PEG modification significantly increased the α-helix and random coil content of HMFP, while the β-sheet and β-turn content decreased. These changes indicate that PEG modification likely facilitated the ordered folding of HMFP, enhancing its structural relaxation and flexibility. This was also validated by the scanning electron microscopy (SEM) results (Figure 5). While the SEM analysis revealed significant morphological changes between HMFP and HMFP-PEG in the dried state, it is important to note that such observations may not fully reflect the protein conformation or interaction behavior in solution. The PEGylation-induced changes in the surface structure may result from altered drying and aggregation properties, rather than direct structural transformation in the aqueous phase.

The thermogravimetric analysis (TGA) and differential thermogravimetric (DTG) analysis further confirmed the impact of PEG modification on the thermal stability of HMFP (Figure 4). In the low-temperature region (around 190 °C), PEG modification may have affected the intramolecular interactions within the protein, making certain parts of its structure more prone to degradation. In the high-temperature region (around 311 °C), the protective effect of PEG likely reduced the thermal stability of HMFP. These changes are consistent with the FTIR results, suggesting that PEG modification modulates the protein’s structure, affecting its thermal stability and degradation behavior.

HMFP, as a naturally derived biomacromolecule, possesses unique antioxidant properties, making it a promising candidate in biomedical applications, particularly in cell protection and oxidative stress defense. Among its components, DOPA stands out due to its strong antioxidant characteristics, as it can scavenge free radicals and stabilize oxidative stress environments, thereby improving cell survival rates [63]. This study demonstrates that both HMFP and HMFP-PEG effectively mitigate hydrogen peroxide (H_2_O_2_)-induced oxidative injury in fibroblasts, and their antioxidant activity is closely linked to the DOPA residues that they contain (Figure 6). In the H_2_O_2_-induced cell injury model, cell viability significantly increased as the concentrations of HMFP and HMFP-PEG increased, with the maximum effect observed at a high concentration (20 μM). Furthermore, HMFP-PEG exhibited a stronger antioxidant capacity at the same concentration, likely due to the enhanced functional stability provided by PEG modification. This suggests that HMFP and HMFP-PEG may alleviate H_2_O_2_-induced cellular injury by directly scavenging free radicals and regulating the redox balance. Additionally, the DPPH radical scavenging experiment further confirmed the antioxidant potential of HMFP and HMFP-PEG, particularly at higher concentrations, where HMFP-PEG displayed superior radical scavenging activity. This highlights the enhanced antioxidant effects of HMFP-PEG, which could potentially provide better protection against oxidative stress in biomedical applications.

Oxidative stress injury is often accompanied by a decrease in the activity of intracellular antioxidant enzymes and the accumulation of lipid peroxidation products [43,64]. In this study, we found that, after H_2_O_2_ induction, the activity of SOD, CAT, and GSH-Px in fibroblasts significantly decreased, while the levels of the lipid peroxidation marker MDA significantly increased (Figure 7). However, pre-treatment with HMFP and HMFP-PEG effectively enhanced the activity of antioxidant enzymes while reducing the MDA levels, indicating that both can modulate the antioxidant enzyme system and enhance the cellular resistance to oxidative stress. Notably, HMFP-PEG showed superior enhancements in the SOD and CAT activity compared to HMFP, which may be attributed to the increased stability of the protein due to the PEG modification, thereby improving its antioxidant effects. Oxidative stress not only affects antioxidant enzyme activity but can also promote cell apoptosis by inducing Bax/Bcl-2 imbalance (Figure 8). This study demonstrates that HMFP and HMFP-PEG can reduce Bax expression while increasing Bcl-2 expression, restoring the balance of apoptosis regulation within the cells. This further confirms their antioxidant-protective role (Figure 9).

The PI3K/Akt signaling pathway plays a crucial role in cell survival and antioxidant defense, with its activation enhancing the cellular antioxidant capacity and reducing oxidative stress injury [46,65,66]. This study demonstrates that pre-treatment with HMFP and HMFP-PEG significantly upregulated the expression levels of p-PI3K and p-Akt, with a more pronounced increase observed in the HMFP-PEG group. This suggests that PEG modification may enhance its ability to activate the PI3K/Akt signaling pathway (Figure 10). Furthermore, the Nrf2/HO-1 signaling pathway also plays a vital role in the oxidative stress response [47]. The Western blot results indicate that both HMFP and HMFP-PEG significantly upregulated the expression of Nrf2 and its downstream target gene HO-1, suggesting that they may enhance the cell’s antioxidant defenses by activating the Nrf2/HO-1 pathway (Figure 11). Notably, the expression levels of Nrf2 and HO-1 in the HMFP-PEG group were higher than those in the HMFP group, further indicating that PEG modification may improve the bioavailability of the protein, thereby enhancing its antioxidant effects.

This study has preliminarily confirmed the antioxidant properties of HMFP and HMFP-PEG. Future research could further explore the in vivo pharmacokinetic characteristics of HMFP-PEG, particularly its long-term stability and biocompatibility. Additionally, while HMFP and HMFP-PEG have demonstrated good antioxidant activity in cell models, their protective effects in animal models and their potential to prevent and treat oxidative stress-related diseases (such as inflammation, cardiovascular diseases, etc.) still require verification. Given its cell-protective effects, HMFP-PEG holds promise for applications in anti-aging, cancer therapy, and wound healing. The further optimization of PEG modification strategies or their combination with anti-inflammatory drugs or chemotherapy agents may enhance their targeting abilities and therapeutic potential, offering new approaches for combination therapies.

## 5. Conclusions

This study is the first to successfully extract water-soluble mussel foot protein (HMFP), synthesize its bifunctional polyethylene glycol-modified form (HMFP-PEG) through a stepwise selective enzymatic hydrolysis method, and verify their antioxidant activity and cell-protective effects.

HMFP and HMFP-PEG may exert antioxidant protection through multiple mechanisms, including radical scavenging, the regulation of antioxidant enzyme activity, the inhibition of cell apoptosis, and the activation of the PI3K/Akt and Nrf2/HO-1 signaling pathways. Notably, HMFP-PEG outperformed HMFP in enhancing the antioxidant capacity and protecting cells, possibly due to the PEG modification, which improved its stability and bioactivity. This study highlights the potential application of mussel foot protein in the antioxidant field, providing scientific evidence for its development and utilization in biomedical materials and tissue engineering.

## Data Availability

Data will be made available on request.
